# Using Ecological Niche Models and Niche Analyses to Understand Speciation Patterns: The Case of Sister Neotropical Orchid Bees

**DOI:** 10.1371/journal.pone.0113246

**Published:** 2014-11-25

**Authors:** Daniel P. Silva, Bruno Vilela, Paulo De Marco, André Nemésio

**Affiliations:** 1 Programa de Pós-Graduação em Ecologia e Evolução, Departamento de Ecologia, ICB, Universidade Federal de Goiás, Rodovia Goiânia-Nerópolis Campus II, Setor Itatiaia, Goiânia, Brazil; 2 Theory, Metapopulation, and Landscape Lab, Departamento de Ecologia, Instituto de Ciências Biológicas, Universidade Federal de Goiás Campus II, Goiânia, Brazil; 3 Instituto de Biologia, Universidade Federal de Uberlândia – UFU, Rua Ceará, Campus Umuarama, Uberlândia, Brazil; Universidade de São Paulo, Brazil

## Abstract

The role of past connections between the two major South American forested biomes on current species distribution has been recognized a long time ago. Climatic oscillations that further separated these biomes have promoted parapatric speciation, in which many species had their continuous distribution split, giving rise to different but related species (i.e., different potential distributions and realized niche features). The distribution of many sister species of orchid bees follow this pattern. Here, using ecological niche models and niche analyses, we (1) tested the role of ecological niche differentiation on the divergence between sister orchid-bees (genera *Eulaema* and *Eufriesea*) from the Amazon and Atlantic forests, and (2) highlighted interesting areas for new surveys. Amazonian species occupied different realized niches than their Atlantic sister species. Conversely, species of sympatric but distantly related *Eulaema* bees occupied similar realized niches. Amazonian species had a wide potential distribution in South America, whereas Atlantic Forest species were more limited to the eastern coast of the continent. Additionally, we identified several areas in need of future surveys. Our results show that the realized niche of Atlantic-Amazonian sister species of orchid bees, which have been previously treated as allopatric populations of three species, had limited niche overlap and similarity. These findings agree with their current taxonomy, which treats each of those populations as distinct valid species.

## Introduction

Past connections between the Amazon and Atlantic forests have long been postulated and phylogenetic relatedness of many taxa occurring in both biomes now support this view [1]–[4]. The taxonomic status of many species in both biomes has long been challenged and different species concepts can be used to support distinct points of view about the taxonomic status of populations occurring in the two biomes [5]–[8].

Both forests were connected during two or three periods in the past [9], [10]. One of those connected areas ranged from western Bolivia, Paraguay, and the state of Mato Grosso to the southeastern region of the state of Paraná in Brazil, after the Andean uplift in the late Miocene (∼23 Mya to 5.6 Mya) [4], [11], [12]. Earlier Pleistocene connections also seem to have existed: one along the Atlantic coast via northeastern Brazil, which today is covered by the ‘Caatinga’ biome, and the other in central Brazil, a region that is now covered by seasonally dry savannas. At the end of the Last Glacial Maximum (*ca.* 5 kya) temperatures rose and humidity decreased, and formed the ‘Caatinga’ and ‘Cerrado’ biomes. Nonetheless, there was biotic interchange between biomes during older periods when both forests were linked. Such similarity has already been shown for several biological groups, such as plants [9], [13], birds [4], [14], butterflies [15], mammals [2], [5], [16], solitary Tapinotaspidini bees [17], and amphibians [18], [19].

During these expansions and contractions, populations of wide-ranged species previously occurring on both areas were separated into two subpopulations that then underwent speciation. At least two mechanisms may explain this process. Following the division and isolation of the original panmictic population, (1) each subpopulation diverged and later evolved into new lineages [20], [21], promoted by the lack of gene flow between both lineages, together with diverging environmental conditions [22], [23]. Conversely, (2) if environmental conditions in both regions remained similar, then the niches of both isolated populations did not change. This process, also known as niche conservatism [24], would lead to a situation in which the isolated populations are less prone to become different species, although, sexual selection may promote speciation, even though sister species share the same environmental niche (e.g., [25]).

Both scenarios seem plausible to Neotropical orchid bees (Hymenoptera: Apidae: Euglossina) associated with forested biomes [26], [27]. Recent studies have pointed out that sister species, with allopatric distributions in these two South American biomes, have obvious affinities (see Table 1) [28]. This likely occurred in recent speciation processes [28]–[38]. However, another line of thought poses that morphological and molecular differences are not consistent and isolated populations should be treated as the same species [39]–[41]. Although morphologically similar, if Atlantic and Amazonian populations are indeed distinct species, their respective Grinnellian niches [42] would be different, mirroring their relationships with environmental conditions in each biome.

**Table 1:** Sister species of orchid bees either occurring in the Amazon or the Atlantic rainforests.

Atlantic Forest	Amazon Forest
***Eufriesea atlantica* Nemésio**	***Eufriesea ornata* (Mócsary)**
*Eufriesea pyrrhopyga* Faria & Melo	*Eufriesea purpurata* (Friese)
*Euglossa adiastola* Hijonosa-Díaz, Nemésio & Engel	*Euglossa augaspis* Dressler
*Euglossa marianae* Nemésio	*Euglossa bidentata* Dressler
*Euglossa cyanochlora* Moure	*Euglossa intersecta* Audouin
*Euglossa botocuda* Faria & Melo	*Euglossa iopyrrha* Dressler
*Euglossa calycina* Faria & Melo	*Euglossa mixta* Friese
*Euglossa bembei* Nemésio	*Euglossa ioprosopa* Dressler
*Euglossa monnei* Nemésio	*Euglossa magnipes* Moure
*Euglossa clausi* Nemésio & Engel	*Euglossa moratoi* Nemésio & Engel
*Euglossa pepei* Nemésio & Engel	*Euglossa parvula* Dressler
***Eulaema niveofasciata* (Friese)**	***Eulaema bombiformis* (Packard)**
***Eulaema atleticana* Nemésio**	***Eulaema meriana* (Olivier)**
*Eulaema felipei* Nemésio	*Eulaema mocsaryi* (Friese)
*Exaerete salsai* Nemésio	*Exaerete trochanterica* Friese

The species analyzed in this study are highlighted in bold.

One way of assessing whether environmental niches of sister species remain conserved after allopatric speciation is using Ecological Niche Models (hereafter ENMs). By using these methods, it is possible not only to evaluate niche dynamics of a species, but also to determine the most important environmental predictors affecting their potential distribution [24], [43], [44]. By assuming niche conservatism between sister species and using ENMs, we expect that the potential distribution of one species would reciprocally predict climatically-suitable areas for the other. If the reverse is true, then niches of both species have already evolved into different environmental requirements after allopatric speciation. Another framework to assess the realized niches of different species, while accounting for inherent biases in occurrence data was proposed by Warren et al. [45] and Broennimann et al. [46]. These methods measure niche overlap between each sister species, comparing it with a randomized distribution. Overlaps larger than expected at random indicate that the sister species occupy similar environmental spaces. Although such methods still consider only a portion of the species' realized niches, they control for unbalanced sampling efforts [46]. Along with environmental input data, these methods also require species occurrences. However, the distributions of insect species is still so fragmentary (the so called Wallacean shortfall; [47], [48]) that the assessment of insect biodiversity and the effectiveness conservation actions involving these organisms is deeply affected, especially at broad spatial scales. Nonetheless, ENMs are important methods to address these issues [47], [48], as well as to inform suitable areas for future field surveys [49].

Given the paleofloristic link between Atlantic and Amazonian rainforests and the controversy related to the taxonomy of Atlantic-Amazonian orchid bees, the main question to be answered in this study regards how much have changes in environmental conditions driven divergence between these lineages. Therefore, we used niche analyses and ENMs to quantify and define the niche features of sister orchid-bee species with allopatric distributions, measuring their niche overlap in an attempt to test whether or not they suffered niche evolution. Additionally, given the overall shortness of knowledge on insect species distribution on the Neotropical region, we used ENMs to predict their potential distributions in both biomes, highlighting relevant areas to future surveys. We expect that realized environmental niches of allopatric and related pairs of Amazonian and Atlantic species from the genera *Eulaema* and *Eufriesea* would show significant divergence with little overlap. Additionally, we also expect that the pairs of allopatric but unrelated *Eulaema* species would have low niche overlaps. Conversely, we expect that the pairs of sympatric and unrelated *Eulaema* species, which remained under the same environmental conditions during their whole evolutionary history, would show a larger environmental niche overlap when compared to pairs of allopatric and related Atlantic-Amazonian sister species. Finally, if the niches of sister species are conserved, they are expected to predict the potential distributions of one another.

## Methods

### Distributional data

We gathered distributional data on orchid-bee species from i) literature records (see Supplementary Material for complete list of published papers); ii) online databases [Species Link (http://splink.cria.org.br), Global Biodiversity Information Facility (http://www.gbif.org)]; and iii) personal collection data from one of us (AN). The species studied had at least 20 unique occurrence records (Table S1). We checked all occurrences regarding their taxonomic reliability, based on the known distribution of the species, and excluded dubious or unreliable records. We used Google Earth [50] to acquire proxy geographical information coordinates for records lacking exact geographic coordinates.

### Environmental layers, modeling procedures, and evaluation

Species occurrences were overlaid in a grid of cells of size of 5 arc-min ranging from south USA to south South America. Using this same grid and considering all 19 bioclimatic variables from WorldClim (http://www.worldclim.org/), we derived principal components (hereafter PCs) to be used as new environmental layers in our distribution models. We selected seven PCs (≈97% of the original climatic variation; Table S2) as our environmental variables.

Given the overall biases and uncertain nature of ENMs, different algorithms may produce divergent species potential distributions [51]. Therefore, we generated the species potential distributions using four different algorithms: i) Envelope Score (ENVSC), a quantitative version of BIOCLIM [52], [53]; ii) Mahalanobis Distance (MAHAL) [54], iii) GARP with best subsets [55], and iv) Maximum Entropy (MAX) [56], [57]. While ENVSC and MAHAL are simpler algorithms that usually need presence data only to produce the species' potential distributions, MAX and GARP are artificial intelligence methods that are generally more complex, and correctly predict the species known occurrences more often [58]. We used the software MaxEnt to run MAX [57], and openModeller Desktop [59] for all the other three modeling algorithms.

We randomly divided all occurrences into ten 70%-30% training-testing subsets. We used the ROC threshold to balance both omission and commission errors while modeling the species distributions [60], to cut the suitability matrices of the modeled species in all modeling algorithms into presence/absence maps. True Skilled Statistics (TSS hereon; [61]), a threshold-dependent statistics, was used to assess model performance. TSS vary from −1 to +1, where negative and around zero values indicate that distributions are no better than random, while values near +1 represent perfect agreement between the observed and the modeled distributions. Acceptable models are those with at least 0.5, and excellent those with TSS around 0.7.

### Quantifying the overlap between pairs of orchid-bee species

We used the same 19 bioclimatic variables from WorldClim in Broennimann et al.'s [46] framework, implemented in R [62], to compare niche features of all six species considered. We used a Principal Components Analysis (PCA) calibrated on the entire environmental space of the study background (Broennimann et al.'s [46] PCA-env) to measure the niche overlap of all species. We compared Atlantic-Amazonian species in pairs (*El. atleticana versus El. meriana*; *El. niveofasciata versus El. bombiformis*; and *Ef. atlantica versus Ef. ornata*). This routine compares the environmental conditions available for species within the full study background with its observed occurrences.

At first, this method encompasses both the niche and the density of occurrences of the species. It calculates the available environmental space, defined by the first two axes from the PCA-env, for each study area. This method corrects for potential sampling bias on occurrence records using a smooth kernel density function [46]. Later, the method obtains an observed niche overlap score for each species pair using Schoener's D metric [63], which varies from 0 to 1, representing a gradient of complete dissimilarity to fully overlapping niches. Then, two different randomization routines are used to test the hypotheses of niche evolution. First, the niche equivalency test compares whether the environmental niche overlap is different from random, while maintaining the original sample sizes. Secondly, the niche similarity test compares the niche overlap of one range randomly distributed over its background keeping the other unchanged (1→2), and then does the reciprocal comparison (1←2). We repeated each randomization process 100 times, producing a null distribution of overlap values to which the observed score was compared. If the observed overlap was significantly smaller, both occurrence sets were different, which would imply that the species were occupying distinct segments of environmental space [46]. Once we had only one pair of sister *Eufriesea* species with a minimum amount of unique occurrences, we did not compare them with other allopatric and unrelated *Eufriesea* sister species.

### Determining priority areas for future surveys of orchid-bee species

After we produced 40 presence/absence maps for each species (10 with each algorithm), we produced a mean consensual distribution map for each species with those which achieved TSS values >0.4. Many orchid bees depend on densely forested areas to occur, since those areas are expected to offer them suitable environmental conditions. Therefore, we used the information on Atlantic Forest remnants (http://www.sosma.org.br/) and the worldwide tree cover data [64] to respectively detect areas with at least 16 km^2^ for the Atlantic (*El. atleticana*, *El. niveofasciata*, and *Ef. atlantica*) and the Amazonian species (*El. bombiformis*, *El. meriana*, and *Ef. ornata*) that are suitable areas for future surveys. The data on forest remnants in the Amazon and the Atlantic forests were converted to raster files with grid cells with 0.041° (≈4 km), allowing us to detect rainforest remnants with at least 16 km^2^, the same resolution to which the final species consensual maps were downscaled. Later, we identified grid cells where each orchid bee was predicted to occur in rainforest remnants with, at least, this minimum area.

## Results

### Orchid bees' realized niche comparisons

**Figure 1:**
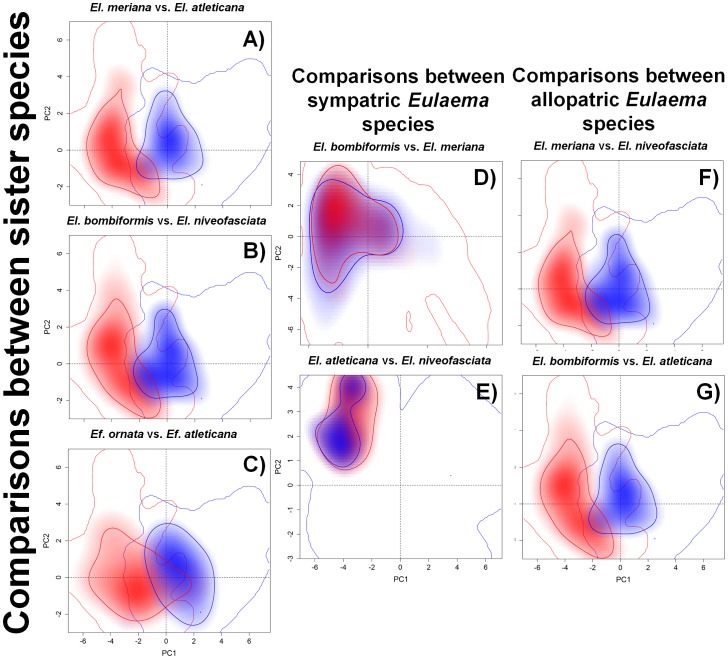
Niche overlap between each pair of Amazonian and the Atlantic sister orchid-bee species obtained through Broennimann et al.'s (2012) framework. A) Niche overlap between the Amazonian *El. meriana* (red) and the Atlantic *El. atleticana* (blue). B) Niche overlap between the Amazonian *El. bombiformis* (red) and the Atlantic *El. niveofasciata* (blue). C) Niche overlap between the Amazonian *Ef. ornata* (red) and the Atlantic *Ef. atlantica* (blue). D) Niche overlap between the sympatric but unrelated Amazonian *Eulamea* species, *El. bombiformis* (red) and *El. meriana* (blue). E) Niche overlap between the sympatric but Atlantic *Eulaema* species, *El. atleticana* (red) and *El. niveofasciata* (blue). F) Niche overlap between the allopatric and unrelated Amazonian *El. meriana* (red) and the Atlantic *El. niveofascita* (blue). G) Niche overlap between the allopatric and unrelated Amazonian *El. bombiformis* (red) and the Atlantic *El. atleticana* (blue). The solid red and blue thin lines correspond to 100% of the available (background) environment for each species considered in the analyses. Red and blue shadings surrounded by thick lines correspond to the density of occurrences of each species per grid cell.

As predicted, both allopatric and unrelated *Eulaema* species and all allopatric sister species from Atlantic-Amazonian forests (*Eufriesea* species included) had small niche overlap. Conversely, sympatric and unrelated *Eulaema* species had a large niche overlap. Generally, Amazonian orchid bees were associated with higher temperatures and less seasonal rainfall than Atlantic species (Fig. 1A–G; Fig. S1). Nevertheless, Amazonian species had a wider climatic range than Atlantic ones (Fig. 1A–C, Fig. S1).

Pairs of sympatric (Fig. 1D–E) and allopatric (Fig. 1F–G) *Eulaema* species had a lower niche overlap and a lower niche equivalency. Niche similarity of *El. atleticana* with its Amazonian sister species, *El. meriana*, was smaller than expected by chance, but the reverse was not true (Table 2; Fig. 1A). Similarly, pairs of allopatric and unrelated *Eulaema* species showed smaller overlaps, and the similarity between Atlantic species and their related allopatric counterparts was also small (Table 2; Fig. 1F–G). Conversely, sympatric but unrelated *Eulaema* species had high overlap of equivalent niches, but their realized niches were less similar (Table 2; Fig. 1D–E), even for sympatric *Eulaema* species.

**Table 2:** Results of the pair-wise niche comparison between the Atlantic-Amazonian orchid-bee species.

Niche comparisons		p Values		
**Atlantic *vs.* Amazonian species**	**D**	**Equivalency**	**Similarity 1→2**	**Similarity 2→1**
*El. atleticana vs. El. meriana*	0.156	**0.019**	0.594	**0.019**
*El. niveofasciata vs. El. bombiformis*	0.160	**0.019**	0.554	0.059
*Ef. atlantica vs. Ef. ornata*	0.140	**0.019**	0.277	0.118
**Sympatric *Eulaema* species**				
*El. bombiformis vs. El. meriana*	0.793	0.059	**0.019**	**0.019**
*El. niveofasciata vs. El. atleticana*	0.678	0.059	**0.019**	**0.019**
**Allopatric *Eulaema* species**				
*El. bombiformis vs. El. atleticana*	0.171	**0.019**	0.693	**0.019**
*El. meriana vs. El. niveofasciata*	0.151	**0.019**	0.475	**0.019**

Bold values are statistically significant. Allopatric comparisons refer to *Eulaema* species from the Atlantic and Amazonian forest, which do not compose a pair of sister species. In the sympatric comparisons, either Atlantic or Amazonian *Eulaema* species are compared. The metric D refers to Schoener's [63] overlap metric, used in the niche comparison analyses proposed by Wareen et al. [45] and Broennimann et al. [46].

### Potential distributions of orchid bees

Regardless of the pair of allopatric species considered, those inhabiting the Atlantic coast always had higher TSS values than Amazonian ones (Table 3). However, TSS values for the *Eufriesea* species were similar, independently of the modeling algorithm considered. In general, TSS values were either acceptable (higher than 0.5) or excellent (higher than 0.7), but the TSS values for *El. meriana*, *Ef. atlantica*, and *Ef. ornata* showed poor prediction rates for ENVSC and MAHAL (Table 3).

**Table 3:** Mean True Skilled Statistic (TSS) values ± their standard deviation obtained for each species in each different modeling algorithms considered in this study.

Species	Envelope score	Mahalanobis distance	GARP	MaxEnt
***El. atleticana***	0.634±0.117	0.760±0.096	0.812±0.084	0.875±0.068
***El. meriana***	0.236±0.016	0.435±0.035	0.506±0.071	0.577±0.037
***El. niveofasciata***	0.718±0.112	0.847±0.095	0.833±0.063	0.905±0.067
***El. bombiformis***	0.551±0.043	0.576±0.051	0.508±0.093	0.588±0.027
***Ef. atlantica***	0.351±0.237	0.421±0.158	0.505±0.109	0.542±0.169
***Ef. ornata***	0.277±0.225	0.428±0.214	0.493±0.208	0.521±0.194

**Figure 2:**
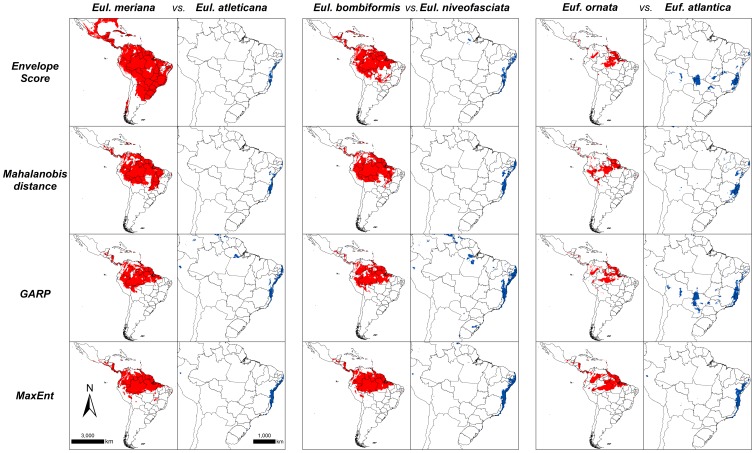
Potential distribution of the modeled *Eulaema* and *Eufriesea* species according to all different modeling algorithms used, and considering both pairs of the Atlantic-Amazonian species evaluated in this study.

**Figure 3:**
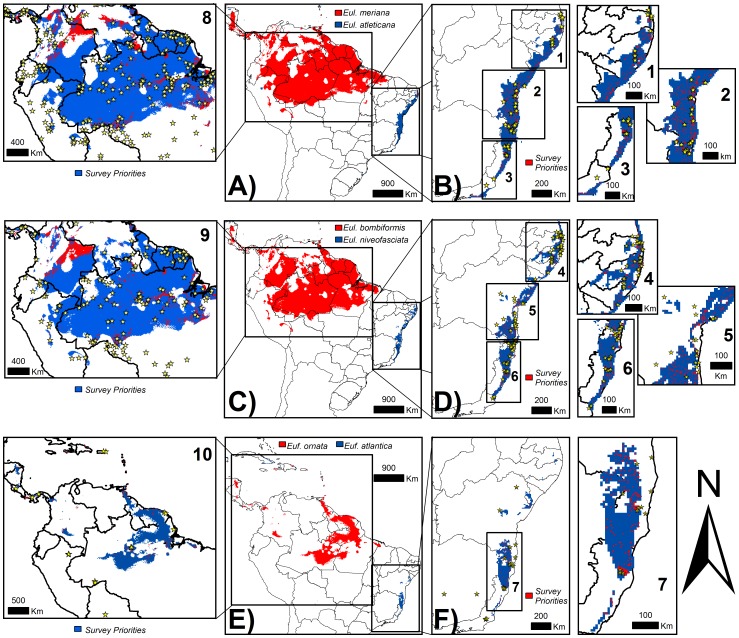
Compared mean consensual distributions of each different pair of Atlantic-Amazonian species considered and future survey priority areas for the Atlantic *Eulaema* species. A) Potential distribution comparison of *El. atleticana* and *El. meriana*. B) Survey priorities for *El. atleticana* in the Brazilian Atlantic coast. C) Potential distribution comparison of *El. bombiformis* and *El. niveofasciata*. D) Survey priorities for *El. niveofasciata* in the Brazilian Atlantic coast. E) Potential distribution comparison of *Ef. ornata* and *Ef. atlantica*. F) Survey priorities for *Ef. atlantica* in the Brazilian Atlantic coast. Stars correspond to the known occurrences of the Atlantic species considered in the ENMs procedures. Inset figures 1–10 highlight priority areas for future field surveys of all the species considered in this study.

While both Amazonian *Eulaema* species showed wider potential distributions, usually ranging from Central America to central South America, the two Atlantic species showed narrower potential distributions, especially along the Atlantic coast in South America (Fig. 2), similarly to *Eufriesea* species. Nonetheless, the distribution of the Amazonian *Ef. ornata* was more constrained than that of the Amazonian species of *Eulaema* (Fig. 2). In general, Atlantic species did not project suitable areas for Amazonian species and the opposite was also true (Fig. 3). Finally, while allopatric species of *Eulaema* did not predict one another potential distributions, the sympatric species did.

Interior areas in the state of Bahia and eastern portions of Sergipe and Alagoas (Fig. 3) were priority areas for future surveys of Atlantic species, since they have large remnants of Atlantic Forest. Since the distribution of *Ef. atlantica* was more constrained than their Atlantic forest relatives, only interior areas in Bahia were indicated as potential for future surveys. Northern Brazil and northern South America (Fig. 3) are priorities areas for future surveys of Amazonian species. Similarly to *Ef. atlantica* in the Atlantic Forest, the constrained potential distribution of *Ef. ornata* in the Amazon revealed areas for future surveys only in northern Brazil, Guianas, and Suriname.

## Discussion

Here, we showed that while allopatric orchid bees had a small niche overlap, an indicative that their niches adapted to different environmental conditions, sympatric species had very similar environmental niches. On the same direction, our ENMs showed that while the pairs of sympatric species predicted suitable climatic conditions for one another, the same did not happen with the pairs of allopatric species: ENMs for Amazonian species did not predict suitable areas for their counterparts in the Atlantic Forest and vice versa. Our results agree with previous studies on niche conservatism of sister species (e.g., [24], [65], [66]). Specifically, we found that sympatric but unrelated *Eulaema* species correctly predicted some portion of the environmental space of the other parapatric congeneric species, which suggests these species likely experienced similar environmental pressures throughout their evolutionary history. Conversely, we found that allopatric but closely-related orchid bees in the Amazon and Atlantic Forest did not predict suitable areas for one another, what challenges both theory and previous studies on niche conservatism [24], [65], [66]. Such patterns also hold if we consider the cross-comparisons of the realized niches of allopatric, sympatric, and sister allopatric orchid-bee species.

The contrasting environmental niches found for the orchid bees from both biomes could be due to varying competition and parasitism rates by other orchid bees [67] or differences in micro-habitat preference in those biomes. Nonetheless, given the spatial scale of our study three other factors seem to be more likely. First, environmental conditions may have been more constant for Amazonian species than in Atlantic Forest. Consequently, Amazonian lineages would not need to adapt to new climatic conditions. As a result, species would have to develop physiological adaptations to cope with the expanding/contracting environmental gradient on the Atlantic coast. Second, natural characteristics of the two biomes, such as a larger area and narrower climate variation in the Amazon could facilitate the spread of Amazonian species. Also, differences in latitudinal and altitudinal variation between the Amazon and the Atlantic forest may to be key elements to explain the range and niche differences. The Atlantic Forest spreads over 27 degrees in latitude [68] and ranges from the sea level up to 2,700 m, whereas the Amazon Basin rarely reaches beyond 1,000 m [69]. Lastly, longitudinal variation is also important to explain such differences, since the dry forests of the inner parts of the Atlantic forest are climatically different from those closer to the coast [70]. Therefore, past cycles of forest expansion and contraction coupled with contrasting environmental conditions in both Amazonian and Atlantic biomes may have forced each subpopulation inhabiting these areas to adapt to different environmental conditions, which later shaped their niches and distributions, promoting their subsequent speciation.

The importance of changing environmental conditions for the divergent speciation of the Atlantic-Amazonian species following forest expansions and contractions were also found by López-Uribe et al. [71], at least for two of the species analyzed (*El. meriana* and *El. bombiformis*). However, it is noteworthy that those authors, without further explanation, considered both Amazonian and Atlantic populations of *El. meriana* and *El. bombiformis* as belonging to the same taxonomic entity, what led them to group all occurrences to generate their ecological niche model, contrarily to the established taxonomy classification that considers them as four different taxa [29], [30], [39], [72], [73], as also supported by our data. Moreover, their own molecular data suggest that Atlantic populations of both *Eulaema* species seem to be monophyletic. Even though these bees have high dispersal rates [74]–[76], López-Uribe et al. [71] showed that their distribution may have been severely affected by South American paleoclimatic instability, which differed between Amazonian and Atlantic forests and supports the niche differences we found for the orchid bees considered in this study. Future studies such as that could be useful to assess how orchid bees responded to past climate changes in South America.

One criticism about niche shifts between related taxa is the use of biased species occurrences data to study realized niche and infer processes driving their fundamental niche. Since species distribution is the intersection between biotic, abiotic, and historically reached habitats, much of the fundamental niche may still not be accessed [42], [77], [78]. Additionally, sampling bias may also prevent the assessment of all reachable areas [42]. Finally, the assumptions of ENMs techniques are subjected to criticisms, since key biological processes (e.g., migration, demographic features, species competition, and predation) are implicit, while a more explicit treatment would be more efficient to assess the species' fundamental niche [79]–[81].

Although our analyses were based on the species realized niches, we believe that our results may help understand speciation patterns of these species. Our results agree with previous studies using the same methods to discuss niche conservatism of exotic species [82], [83] and the division of a single lineage into several ones [84]–[87]. Thus, given the idiosyncratic environmental niches, our results support the splitting of Amazon and Atlantic populations into different taxonomic entities [28]–[38], [88].

The distributions of insect species in the tropics suffers from knowledge gaps [47], [48], specially taxonomic identity (Linnean shortfall) and spatial distribution (Wallacean shortfall). Orchid bees are no exception [89], especially regarding taxonomic issues [90]. Nonetheless, ENMs are important tools to assess the distribution of “data deficient” species [48], allowing them to be included in broad-scale conservation studies. Because orchid bees depend on humid forested areas [26], [27], some have been reported as threatened [91] or extinct in fragmented areas in the Atlantic Forest [28], [33]. Therefore, integrating ENMs and information on original vegetation remnants are urging. Given this scenario, future surveys in the Amazonian and Atlantic Forest fragments highlighted by our analyses may add important information on species distribution and their natural history.

## Conclusions

In this study, based in SDMs and analyses of the species' realized niches in South America, we have shown that sister Atlantic vs. Amazonian orchid bees, namely *El. atleticana* vs. *El. meriana*, *El. bombifromis* vs. *El. niveofasciata*, and *Ef. atlantica* vs. *Ef. ornata*, had different potential distributions and realized niche features. Nonetheless, we also showed that sympatric species have more similar environmental niches than that observed for the pairs of sister Atlantic-Amazonian orchid bees. These results support the view that the orchid bee sister lineages from Amazonian and Atlantic forests are currently under divergent selection, and consequently should be treated as different species. Additionally, we also highlighted important areas to be considered in future surveys in both biomes. We believe that similar approaches to those we used here are important to unveil both the biogeographic history of the South American fauna, as well as to optimize and guide future biological surveys of these species in the continent. Finally, as already developed for *El. meriana* and *El. bombiformis*, we recommend that future studies should also employ phylogeographic analyses of these (and other) orchid-bee species so we could better understand how past range expansion and contraction events in the continent may have originated the faunal and floral similarities currently found in those biomes.

## Supporting Information

Figure S1 **Results obtained from the PCA-env approach developed by Broennimann et al. (2012).** A) PCA-env obtained with WorldClim's 19 raw environmental variables, considering the whole extent of South and Central Americas, used as the background in the analysis. PC percentages refer to the amount of variation explained by each PCA axis. B) and C) Contributions of each environmental variable to the first and second PCA axes, respectively.
(TIF)

Table S1 **Amount of unique occurrences gathered for each orchid-bee species used in this study.**
(DOC)

Table S2 **Summary of the Principal Component Analysis which generated the principal components (PC) used as environmental layers.** Each cell value represents the individual loadings of each variable in each of the PCs. The PCs, individual, and accumulated proportions of each PCs are also shown.
(DOC)

## References

[1] De Vivo M (1997) Mammalian evidence of historical ecological change in the Caatinga semiarid vegetation of northeastern Brazil. J Comp Biol 2:65–73.

[2] Costa LP (2003) The historical bridge between the Amazon and the Atlantic Forest of Brazil: a study of molecular phylogeography with small mammals. J Biogeogr 30:71–86.

[3] De Vivo M, Carmignotto AP (2004) Holocene vegetation change and the mammal faunas of South America and Africa. J Biogeogr 31:943–957.

[4] Batalha-Filho H, Fjeldså J, Fabre PH, Miyaki CY (2012) Connections between the Atlantic and the Amazonian forest avifaunas represent distinct historical events. J Ornithol 154:41–50.

[5] Simpson GC (1969) South American mammals. In: Fittkau EJ, Illies J, Klinge H, editors. Biogeography and ecology in South America.Vol. 1. The Hague. pp. 113–134.

[6] Vanzolini PE, Williams EE (1970) South American anoles: the geographic differentiation and evolution of the *Anolis chrysolepis* species group (Sauria, Iguanidae). Arq Zool do Mus Zool da Univ São Paulo 19:1–298.

[7] Prance GT (1973) Phytogeographic support for the theory of Pleistocene forest refuges in the Amazon Basin, based on evidence from distribution patterns in Caryocaraceae, Chrysobalaceae, Dichapetalaceae and Lecythidaceae. Acta Amaz 3:5–28.

[8] Prance GT (1979) The taxonomy and phytogeography of the Clirysobalanaceae of the Atlantic coastal forests of Brazil. Rev Bras Botânica 2:19–39.

[9] Méio BB, Freitas CV, Jatobá L, Silva MEF, Ribeiro RPB (2003) Influência da flora das florestas Amazônica e Atlântica na vegetação do cerrado *sensu stricto* . Rev Bras Botânica 26:437–444.

[10] De Oliveira PE, Magno A, Suguio K (1999) Late Pleistocene/Holocene climatic and vegetational history of the Brazilian Caatinga: the fossil dunes of the middle São Francisco River. Palaeogeogr Palaeoclimatol Palaeoecol 152:319–337.

[11] Hoorn C, Wesselingh FP, Steege HT, Bermudez MA, Mora A, et al (2010) Amazonia through time: Andean uplift, climate change, landscape evolution, and biodiversity. Science 330:927–931. 21071659 10.1126/science.1194585

[12] Wesselingh FP, Salo JA (2006) A Miocene perspective on the evolution of the Amazonian biota. Scr Geol 133:439–458.

[13] Oliveira-Filho AT, Ratter JA (1995) A study of the origin of central Brazilian forests by the analysis of plant species distribution patterns. Edinburgh J Bot 52:141–194.

[14] Silva JMC (1996) Distribution of Amazonian and Atlantic birds in gallery forests of the Cerrado region, South America. Ornitol Neotrop 7:1–18.

[15] Brown KS Jr (1987) Biogeography and evolution of Neotropical butterflies. In: Whitmore T, Prance G, editors.Biogeography and Quaternary History in Tropical America.Oxford: Oxford University Press. pp. 66–104.

[16] Redford KH, da Fonseca GAB (1986) The role of gallery forests in the zoogeography of the Cerrado's non-volant mammalian fauna. Biotropica 18:126–135.

[17] Aguiar AJC, Melo GAR (2007) Taxonomic revision, phylogenetic analysis, and biogeography of the bee genus *Tropidopedia* (Hymenoptera, Apidae, Tapinotaspidini). Zool J Linn Soc 151:511–554.

[18] Fouquet A, Recoder R, Teixeira M, Cassimiro J, Amaro RC, et al (2012) Molecular phylogeny and morphometric analyses reveal deep divergence between Amazonia and Atlantic Forest species of Dendrophryniscus. Mol Phylogenet Evol 62:826–838. 22166838 10.1016/j.ympev.2011.11.023

[19] Fouquet A, Loebmann D, Castroviejo-Fisher S, Padial JM, Orrico VGD, et al (2012) From Amazonia to the Atlantic forest: molecular phylogeny of Phyzelaphryninae frogs reveals unexpected diversity and a striking biogeographic pattern emphasizing conservation challenges. Mol Phylogenet Evol 65:547–561. 22842094 10.1016/j.ympev.2012.07.012

[20] Wiens JJ (2004) What is speciation and how should we study it? Am Nat 163:914–923. 15266388 10.1086/386552

[21] Wiley EO (1981) Phylogenetics—The Theory and Practice of Phylogenetic Systematics. 1st ed. New York: John Wiley & Sons, Inc.

[22] Schluter D (2001) Ecology and the origin of species. Trends Ecol Evol 16:372–380. 11403870 10.1016/s0169-5347(01)02198-x

[23] Turelli M, Barton NH, Coyne JA (2001) Theory and speciation. Trends Ecol Evol 16:330–343. 11403865 10.1016/s0169-5347(01)02177-2

[24] Wiens JJ, Graham CH (2005) Niche conservatism: Integrating evolution, ecology, and conservation biology. Annu Rev Ecol Evol Syst 36:519–539.

[25] Wellenreuther M, Larson KW, Svensson EI (2012) Climatic niche divergence or conservatism? Environmental niches and range limits in ecologically similar damselflies. Ecology 93:1353–1366. 22834376 10.1890/11-1181.1

[26] Dressler RL (1982) Biology of the orchid bees (Euglossini). Annu Rev Ecol Syst 13:373–394.

[27] Roubik DW, Hanson PE (2004) Orchid bees of tropical America: Biology and field guide. 1st ed. San José: INBio.

[28] Nemésio A (2010) *Eulaema (Apeulaema) felipei* sp. n. (Hymenoptera: Apidae: Euglossina): a new forest-dependent orchid bee found at the brink of extinction in northeastern Brazil. Zootaxa 62:51–62.

[29] Moure JS (2003) As espécies do gênero *Eulaema* Lepeletier, 1841 (Hymenoptera, Apidae, Euglossinae). Acta Biol Parana 29:1–70.

[30] Nemésio A (2009) Orchid bees (Hymenoptera: Apidae) of the Brazilian Atlantic forest. Zootaxa 2041:1–242.

[31] Nemésio A (2011) *Exaerete salsai* sp. n. (Hymenoptera: Apidae): a new orchid bee from eastern Brazil. Zootaxa 2967:12–20.

[32] Nemésio A (2011) *Euglossa bembei* sp. n. (Hymenoptera: Apidae): a new orchid bee from the Brazilian Atlantic Forest belonging to the Euglossa cybelia Moure, 1968 species group. Zootaxa 3006:43–49.

[33] Nemésio A (2011) *Euglossa marianae* sp. n. (Hymenoptera: Apidae): a new orchid bee from the Brazilian Atlantic Forest and the possible first documented local extinction of a forest-dependent orchid bee. Zootaxa 2892:59–68.

[34] Nemésio A (2012) Species of *Euglossa* Latreille, 1802 (Hymenoptera: Apidae: Euglossina) belonging to the purpurea species group occurring in eastern Brazil, with description of Euglossa monnei sp. n. Zootaxa 3151:35–52.

[35] Faria LRR, Melo GAR (2011) A new species of *Eufriesea* Cockerell (Hymenoptera, Apidae, Euglossina) from northeastern Brazil. Rev Bras Entomol 55:35–39.

[36] Faria LRR, Melo GAR (2012) Species of *Euglossa* of the analis group in the Atlantic forest (Hymenoptera, Apidae). Zoologia 29:349–374.

[37] Hinojosa-Díaz IA, Nemésio A, Engel MS (2012) Two new species of *Euglossa* from South America, with notes on their taxonomic affinities (Hymenoptera, Apidae). Zookeys 79:63–79. 10.3897/zookeys.221.3659 PMC3487635 23129981

[38] Nemésio A, Engel MS (2012) Three new cryptic species of *Euglossa* from Brazil (Hymenoptera, Apidae). Zookeys 222:47–68. 10.3897/zookeys.222.3382 PMC3459030 23129986

[39] Dressler RL (1979) *Eulaema bombiformis*, *E. meriana*, and mullerian mimicry in related species (Hymenoptera: Apidae). Biotropica 11:144–151.

[40] Oliveira ML (2006) Três novas espécies de abelhas da Amazônia pertencentes ao gênero *Eulaema* Lepeletier, 1841 (Hymenoptera: Apidae: Euglossini). Acta Amaz 36:121–128.

[41] Oliveira ML (2008) Catálogo comentado das espécies de abelhas do gênero *Eulaema* Lepeletier, 1841 (Hymenoptera: Apidae). Lundiana 8:113–136.

[42] Soberón J (2007) Grinnellian and Eltonian niches and geographic distributions of species. Ecol Lett 10:1115–1123. 17850335 10.1111/j.1461-0248.2007.01107.x

[43] Guisan A, Zimmermann NE (2000) Predictive habitat distribution models in ecology. Ecol Modell 135:147–186.

[44] Pearman PB, Guisan A, Broennimann O, Randin CF (2008) Niche dynamics in space and time. Trends Ecol Evol 23:149–158. 18289716 10.1016/j.tree.2007.11.005

[45] Warren DL, Glor RE, Turelli M (2008) Environmental niche equivalency versus conservatism: quantitative approaches to niche evolution. Evolution 62:2868–2883. 18752605 10.1111/j.1558-5646.2008.00482.x

[46] Broennimann O, Fitzpatrick MC, Pearman PB, Petitpierre B, Pellissier L, et al (2012) Measuring ecological niche overlap from occurrence and spatial environmental data. Glob Ecol Biogeogr 21:481–497.

[47] Whittaker RJ, Araújo MB, Jepson P, Ladle RJ, Watson JEMA, et al (2005) Conservation biogeography: Assessment and prospect. Divers Distrib 11:3–23.

[48] Diniz-Filho JAF, De Marco P Jr, Hawkins BA (2010) Defying the curse of ignorance: Perspectives in insect macroecology and conservation biogeography. Insect Conserv Divers 3:172–179.

[49] Silva DP, Aguiar AJC, Melo GAR, Anjos-Silva EJ, De Marco P Jr (2013) Amazonian species within the Cerrado savanna: new records and potential distribution for *Aglae caerulea* (Apidae: Euglossini). Apidologie 44:673–683.

[50] Google Inc**.** (2013) Google Earth, version 7.0.3.8542.

[51] Diniz-Filho JAF, Bini LM, Rangel TFLVB, Loyola RD, Hof C, et al (2009) Partitioning and mapping uncertainties in ensembles of forecasts of species turnover under climate change. Ecography 32:897–906.

[52] Nix HA (1986) A biogeographic analysis of Australian elapid snakes. In: Longmore R, editor.Atlas of Elapid Snapkes of Australia - Australian Flora and Fauna series Number 7.Canberra: Australian Government Publishing Service. pp. 4–15.

[53] Piñero R, Aguilar JF, Munt DD, Feliner GN (2007) Ecology matters: Atlantic-Mediterranean disjunction in the sand-dune shrub *Armeria pungens* (Plumbaginaceae). Mol Ecol 16:2155–2171. 17498238 10.1111/j.1365-294X.2007.03280.x

[54] Farber O, Kadmon R (2003) Assessment of alternative approaches for bioclimatic modeling with special emphasis on the Mahalanobis distance. Ecol Modell 160:115–130.

[55] Stockwell D, Peters D (1999) The GARP modelling system: problems and solutions to automated spatial prediction. Int J Geogr Inf Sci 13:143–158.

[56] Phillips SJ, Dudik M (2008) Modeling of species distributions with Maxent: new extensions and a comprehensive evaluation. Ecography 31:161–175.

[57] Phillips SJ, Anderson RP, Schapire RE (2006) Maximum entropy modeling of species geographic distributions. Ecol Modell 190:231–259.

[58] Rangel TF, Loyola RD (2012) Labeling ecological niche models. Nat Conserv 10:119–126.

[59] Muñoz MES, De Giovanni R, de Siqueira MF, Sutton T, Brewer P, et al (2011) openModeller: a generic approach to species' potential distribution modelling. Geoinformatica 15:111–135.

[60] Liu CR, Berry PM, Dawson TP, Pearson RG (2005) Selecting thresholds of occurrence in the prediction of species distributions. Ecography 28:385–393.

[61] Allouche O, Tsoar A, Kadmon R (2006) Assessing the accuracy of species distribution models: Prevalence, Kappa and the True Skill Statistic (TSS). J Appl Ecol 43:1223–1232.

[62] R Development Core Team (2013) R: A language and environment for statistical computing. R Foundation for Statistical Computing.

[63] Schoener TW (1970) Nonsynchronous spatial overlap of lizards in patchy habitats. Ecology 51:408–418.

[64] Hansen MC, Potapov PV, Moore R, Hancher M, Turubanova SA, et al (2013) High-resolution global maps of 21st-century forest cover change. Science 342:850–853. 24233722 10.1126/science.1244693

[65] Graham CH, Ron SR, Santos JC, Schneider CJ (2004) Integrating phylogenetics and environmental niche models to explore speciation mechanisms in dendrobatid frogs. Evolution 58:1781–1793. 15446430 10.1111/j.0014-3820.2004.tb00461.x

[66] Peterson AT, Soberón J, Sánchez-Cordero V (1999) Conservatism of ecological niches in evolutionary time. Science 285:1265–1267. 10455053 10.1126/science.285.5431.1265

[67] Nemésio A, Silveira FA (2006) Deriving ecological relationships from geographical correlations between host and parasitic species: An example with orchid bees. J Biogeogr 33:91–97.

[68] Silva JMC, Casteleti CHM (2003) Status of the Biodiversity of the Atlantic Forest of Brazil. In: Galindo-Leal C, Câmara IG, editors.The Atlantic Forest of South America – biodiversity status, threats, and outlook.Washington: Island Press. pp. 43–59.

[69] Câmara IG (2003) Brief history of conservation in the Atlantic Forest. In: Galindo-Leal C, Câmara IG, editors.The Atlantic Forest of South America – biodiversity status, threats, and outlook.Washington: Island Press. pp. 31–42.

[70] Rizzini CT (1997) Tratado de fitogeografia do Brasil: aspectos ecológicos, sociológicos e florísticos. 2nd ed. Rio de Janeiro: Âmbito Cultural Edições.

[71] López-Uribe MM, Zamudio KR, Cardoso CF, Danforth BN (2014) Climate, physiological tolerance and sex-biased dispersal shape genetic structure of Neotropical orchid bees. Mol Ecol 23:1874–1890. 24641728 10.1111/mec.12689

[72] Nemésio A, Rasmussen C (2011) Nomenclatural issues in the orchid bees (Hymenoptera: Apidae: Euglossina) and an updated catalogue. Zootaxa 42:1–42.

[73] Moure JS, Melo GAR, Faria LRR Jr (2007) Euglossini Latreille, 1802. In: Moure JS, Urban D, Melo GAR, editors. Catalogue of Bees (Hymenoptera, Apoidea) in the Neotropical Region - on-line version. Available at http://www.moure.cria.org.br/catalogue. Accessed on 23rd, July, 2014. Curitiba: Sociedade Brasileira de Entomologia. pp. 214–255.

[74] Janzen DH (1971) Euglossine bees as long-distance pollinators of tropical plants. Science 171:203–205. 17751330 10.1126/science.171.3967.203

[75] Raw A (1989) The dispersal of Euglossine bees between isolated patches of eastern Brazilian wet forest (Hymenoptera, Apidae). Rev Bras Entomol 33:103–107.

[76] Wikelski M, Moxley J, Eaton-Mordas A, López-Uribe MM, Holland R, et al (2010) Large-range movements of Neotropical orchid bees observed via radio telemetry. PLoS One 5:e10738. 20520813 10.1371/journal.pone.0010738 PMC2877081

[77] Peterson AT (2011) Ecological niche conservatism: a time-structured review of evidence. J Biogeogr 38:817–827.

[78] Peterson AT, Soberón J, Pearson RG, Anderson RP, Martínez-Meyer E, et al**.** (2011) Ecological niches and geographic distributions. 1st ed. Princeton: Princeton University Press.

[79] McInerny GJ, Etienne RS (2012) Ditch the niche - is the niche a useful concept in ecology or species distribution modelling? J Biogeogr 39:2096–2102.

[80] McInerny GJ, Etienne RS (2012) Stitch the niche - a practical philosophy and visual schematic for the niche concept. J Biogeogr 39:2103–2111.

[81] McInerny GJ, Etienne RS (2012) Pitch the niche - taking responsibility for the concepts we use in ecology and species distribution modelling. J Biogeogr 39:2112–2118.

[82] Petitpierre B, Kueffer C, Broennimann O, Randin C, Daehler C, et al (2012) Climatic niche shifts are rare among terrestrial plant invaders. Science 335:1344–1348. 22422981 10.1126/science.1215933

[83] Strubbe D, Broennimann O, Chiron F, Matthysen E (2013) Niche conservatism in non-native birds in Europe: niche unfilling rather than niche expansion. Glob Ecol Biogeogr 22:962–970.

[84] Wielstra B, Beukema W, Arntzen JW, Skidmore AK, Toxopeus AG, et al (2012) Corresponding mitochondrial DNA and niche divergence for crested newt candidate species. PLoS One 7:e46671. 23029564 10.1371/journal.pone.0046671 PMC3460878

[85] Martínez-Gordillo D, Rojas-Soto O, de los Monteros AE (2010) Ecological niche modelling as an exploratory tool for identifying species limits: an example based on Mexican muroid rodents. J Evol Biol 23:259–270. 20002252 10.1111/j.1420-9101.2009.01897.x

[86] Raxworthy CJ, Ingram CM, Rabibisoa N, Pearson RG (2007) Applications of ecological niche modeling for species delimitation: A review and empirical evaluation using day geckos (Phelsuma) from Madagascar. Syst Biol 56:907–923. 18066927 10.1080/10635150701775111

[87] Tocchio LJ, Gurgel-Gonçalves R, Escobar LE, Peterson AT (2014) Niche similarities among white-eared opossums (Mammalia, Didelphidae): Is ecological niche modelling relevant to setting species limits? Zool Scr. doi:10.1111/zsc.12082

[88] Nemésio A (2007) *Eufriesea atlantica* sp. n. (Hymenoptera: Apidae), a new orchid bee from the Brazilian Atlantic Forest. Lundiana 8:147–152.

[89] Nemésio A (2013) Are orchid bees at risk? First comparative survey suggests declining populations of forest-dependent species. Brazilian J Biol 73:367–374. 10.1590/S1519-69842013000200017 23917564

[90] Nemésio A (2012) Methodological concerns and challenges in ecological studies with orchid bees (Hymenoptera: Apidae: Euglossina). Biosci J 28:118–135.

[91] Nemésio A, Cerântola NCM, Vasconcelos HL, Nabout JC, Silveira FA, et al (2012) Searching for *Euglossa cyanochlora* Moure, 1996 (Hymenoptera: Apidae), one of the rarest bees in the world. J Insect Conserv 16:745–755.

