# Supplementary figures and images for: Using Ecological Niche Models and Niche Analyses to Understand Speciation Patterns: The Case of Sister Neotropical Orchid Bees
Source: PLoS One. 2014 Nov 25;9(11):e113246. doi: 10.1371/journal.pone.0113246 (PMC4244149; doi:10.1371/journal.pone.0113246)

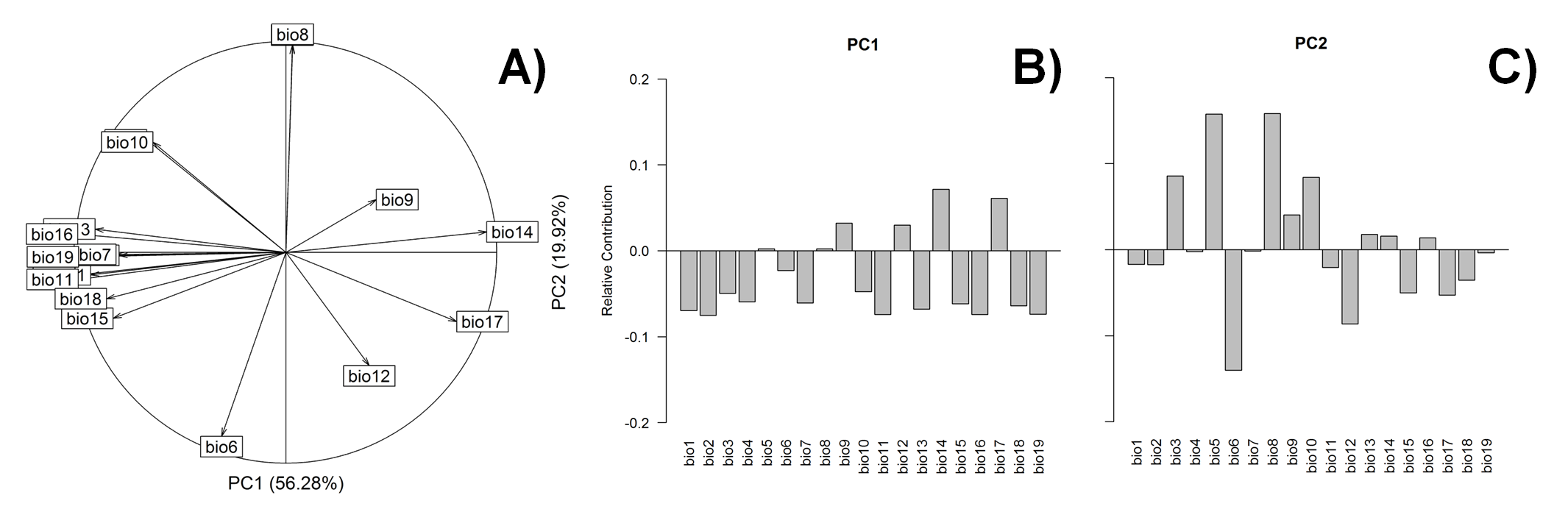

Supplement: Figure S1 — Results obtained from the PCA-env approach developed by Broennimann et al. (2012). A) PCA-env obtained with WorldClim's 19 raw environmental variables, considering the whole extent of South and Central Americas, used as the background in the analysis. PC percentages refer to the amount of variation explained by each PCA axis. B) and C) Contributions of each environmental variable to the first and second PCA axes, respectively. (TIF) [file pone.0113246.s001.tif]
