# Supplementary material for: Using Ecological Niche Models and Niche Analyses to Understand Speciation Patterns: The Case of Sister Neotropical Orchid Bees
Source: PLoS One. 2014 Nov 25;9(11):e113246. doi: 10.1371/journal.pone.0113246 (PMC4244149; doi:10.1371/journal.pone.0113246)
Supplement: Table S1 — Amount of unique occurrences gathered for each orchid-bee species used in this study. (DOC) [file pone.0113246.s002.doc]

**Table S1 – Amount of unique occurrences gathered for each orchid-bee species used in this study**

| **Species** | **Unique occurrence records** |
| --- | --- |
| *Eulaema atleticana* | 37 |
| *Eulaema meriana* | 337 |
| *Eulaema niveofasciata* | 70 |
| *Eulaema bombiformis* | 179 |
| *Eufriesea atlantica* | 21 |
| *Eufriesea ornata* | 20 |
