# Supplementary material for: Using Ecological Niche Models and Niche Analyses to Understand Speciation Patterns: The Case of Sister Neotropical Orchid Bees
Source: PLoS One. 2014 Nov 25;9(11):e113246. doi: 10.1371/journal.pone.0113246 (PMC4244149; doi:10.1371/journal.pone.0113246)
Supplement: Table S2 — Summary of the Principal Component Analysis which generated the principal components (PC) used as environmental layers. Each cell value represents the individual loadings of each variable in each of the PCs. The PCs, individual, and accumulated proportions of each PCs are also shown. (DOC) [file pone.0113246.s003.doc]

**Table S2 -** Summary of the Principal Component Analysis which generated the principal components (PC) used as environmental layers. Each cell value represents the individual loadings of each variable in each of the PCs. The PCs, individual, and accumulated proportions of each PCs are also show

|  | **Principal Components** | | | | | | |
| --- | --- | --- | --- | --- | --- | --- | --- |
| **Variable** | **PC1** | **PC2** | **PC3** | **PC4** | **PC5** | **PC6** | **PC7** |
| Annual mean temperature (Bio 1) | 0.269 | 0.255 | -0.096 | 0.064 | -0.072 | 0.017 | 0.000 |
| Mean diurnal range (Bio 2) | -0.204 | 0.206 | -0.067 | -0.494 | 0.0865 | 0.481 | -0.364 |
| Isotermality (Bio 3) | 0.242 | -0.000 | 0.332 | -0.054 | -0.063 | 0.517 | -0.193 |
| Temperature seasonality (Bio 4) | -0.245 | 0.032 | -0.394 | -0.021 | 0.1649 | -0.110 | 0.140 |
| Maximum temperature warmest period (Bio 5) | 0.136 | 0.369 | -0.344 | -0.026 | 0.0996 | 0.0430 | -0.099 |
| Minimum temperature coldest period (Bio 6) | 0.300 | 0.120 | 0.064 | 0.1815 | -0.078 | -0.006 | 0.019 |
| Temperature anual range (Bio 7) | -0.254 | 0.119 | -0.316 | -0.229 | 0.161 | 0.037 | -0.092 |
| Mean temperature wettest quarter (Bio 8) | 0.205 | 0.305 | -0.184 | -0.064 | -0.194 | 0.042 | 0.354 |
| Mean temperature driest quarter (Bio 9) | 0.268 | 0.178 | -0.017 | 0.163 | 0.101 | 0.053 | -0.270 |
| Mean temperature warmest quarter (Bio 10) | 0.188 | 0.317 | -0.327 | 0.079 | 0.024 | -0.034 | 0.0679 |
| Mean temperature coldest quarter (Bio 11) | 0.292 | 0.183 | 0.064 | 0.064 | -0.103 | 0.054 | -0.055 |
| Annual precipitation (Bio 12) | 0.275 | -0.199 | -0.078 | -0.209 | 0.137 | -0.104 | -0.110 |
| Precipitation wettest period (Bio 13) | 0.279 | -0.076 | 0.062 | -0.308 | 0.244 | -0.290 | -0.086 |
| Precipitation driest period (Bio 14) | 0.151 | -0.355 | -0.286 | -0.000 | -0.106 | 0.354 | 0.215 |
| Precipitation seasonality (Bio 15) | -0.039 | 0.293 | 0.383 | -0.385 | 0.119 | 0.096 | 0.650 |
| Precipitation wettest quarter (Bio 16) | 0.280 | -0.085 | 0.058 | -0.305 | 0.239 | -0.275 | -0.121 |
| Precipitation driest quarter (Bio 17) | 0.162 | -0.353 | -0.284 | -0.005 | -0.079 | 0.319 | 0.185 |
| Precipitation warmest quarter (Bio 18) | 0.173 | -0.184 | -0.151 | -0.483 | -0.528 | -0.203 | 0.000 |
| Precipitation coldest quarter (Bio 19) | 0.211 | -0.204 | -0.064 | 0.070 | 0.638 | 0.164 | 0.214 |
| **Proportion explained by each PC** | 51.855 | 20.316 | 11.959 | 5.553 | 3.964 | 2.483 | 1.416 |
| **Accumulated variation proportion** | 51.855 | 72.171 | 84.131 | 89.684 | 93.649 | 96.132 | 97.548 |
| **Principal Components eigenvalues** | 9.852 | 3.860 | 2.272 | 1.055 | 0.753 | 0.471 | 0.269 |
